# Silencing of Exosomal miR-181a Reverses Pediatric Acute Lymphocytic Leukemia Cell Proliferation

**DOI:** 10.3390/ph13090241

**Published:** 2020-09-11

**Authors:** Shabirul Haque, Sarah R. Vaiselbuh

**Affiliations:** 1 Feinstein Institute for Medical Research, Northwell Health, 350 Community Drive, Manhasset, NY 11030, USA; svaiselbuh1@pride.hofstra.edu; 2 Department of Pediatrics, Staten Island University Hospital, Northwell Health, 475 Seaview Ave, Staten Island, NY 10305, USA

**Keywords:** exosomes, leukemia, miRNA silencing

## Abstract

Exosomes are cell-generated nano-vesicles found in most biological fluids. Major components of their cargo are lipids, proteins, RNA, DNA, and non-coding RNAs. The miRNAs carried within exosomes reveal real-time information regarding disease status in leukemia and other cancers, and therefore exosomes have been studied as novel biomarkers for cancer. We investigated the impact of exosomes on cell proliferation in pediatric acute lymphocytic leukemia (PALL) and its reversal by silencing of exo-miR-181a. We isolated exosomes from the serum of PALL patients (Exo-PALL) and conditioned medium of leukemic cell lines (Exo-CM). We found that Exo-PALL promotes cell proliferation in leukemic B cell lines by gene regulation. This exosome-induced cell proliferation is a precise event with the up-regulation of proliferative (PCNA, Ki-67) and pro-survival genes (MCL-1, and BCL2) and suppression of pro-apoptotic genes (BAD, BAX). Exo-PALL and Exo-CM both show over expression of miR-181a compared to healthy donor control exosomes (Exo-HD). Specific silencing of exosomal miR-181a using a miR-181a inhibitor confirms that miR-181a inhibitor treatment reverses Exo-PALL/Exo-CM-induced leukemic cell proliferation in vitro. Altogether, this study suggests that exosomal miR-181a inhibition can be a novel target for growth suppression in pediatric lymphatic leukemia.

## 1. Introduction

Exosomes are spherical nanoparticles (30–150 nm) produced by normal and pathological cells in most body fluids such as serum, urine, breast milk, and ascites. Exosomes originate from the cytoplasm of the cell by inward budding of multivesicular bodies [1]. Consequently, exosomal content is enriched for proteins, lipids, cytoplasmic mRNA, and most importantly, non-coding micro-RNA [2,3]. Several investigators have established that biologically functional microRNAs (miRNAs) are transported by exosomes and exosome-encapsulated miRNAs can be delivered to target cells, thereby transferring the miRNA-signature from their parental cell of origin. The exosomal cargo that is transferred as such is functionally active, enabling the target cells to reprogram and redefine their immunological responses at a molecular level [4,5,6]. Moreover, exosomes derived from the tumor microenvironment can recruit and impair normal cells to contribute to the malignancy. Some reports in the literature support the fact that miRNAs carried within exosomes reveal real-time information regarding disease status in leukemia and other cancers. Therefore, exosomes have been studied for their potential as novel biomarkers for cancer. The easily accessible in the serum of patients by minimally invasive techniques such as a simple phlebotomy [7,8,9,10]. Our work focuses on the functional role of exosomal-miRNA-181a (exo-miR-181a) in pediatric acute lymphocytic leukemia (PALL).

The miRNAs are endogenously expressed non-coding regulatory RNAs of 20–25 nucleotides long and play a major role as gene expression regulators. They can act as gene expression silencers at a post-transcriptional level either by inhibiting mRNA translation or by mRNA degradation. Furthermore, miRNAs can destabilize the mRNA transcript through binding to the 5′ or 3′ untranslated regions (UTR) [11,12,13]. As such, miRNAs function as facilitators of fundamental normal biological processes such as cell differentiation, proliferation, apoptosis, and survival by manipulation of target genes [14]. Dysregulation of these gene functions may be one of the contributing pathogenic processes that can lead to leukemogenesis [15]. Precisely, the miR-181 family consists of four mature individual miRs, namely miR-181a, miR-181b, miR-181c, and miR-181d, which are highly conserved across almost all vertebrates, suggestive of their functional relevance [16]. Several investigators have established that miR-181a specifically, modulates cellular events at multiple levels—such as cell proliferation, growth, survival and even chemo-sensitivity in cancer [17,18]. Yang et al. described that elevated expression of miR-181a leads to cancer progression and is associated with poor survival, implying a clinical significance role for miR-181a [19,20,21,22]. In contrast, others have shown that down-regulated expression of miR-181a leads to poor prognosis, metastasis, and cancer development [23,24]. This dual behavior of miR-181a has been reported in different types of cancer, suggesting that the mechanism by which miR-181a exerts its functional role might be very disease-specific. There are only a few reports in the literature that studied the role of cellular miR-181a in the proliferation of PALL. To the best of our knowledge, no one has investigated the role of exosomal miR-181a in PALL progression to date. Acute lymphoblastic leukemia (ALL) is one of the most common hematological cancers impacting both adults and children [25]. The peak age of pediatric ALL (PALL) incidence is between 2–4 years [26]. Epigenetic factors, such as DNA methylation, gene regulation and chromatin remodeling, play a significant role in PALL development and progression by dysregulation of miRNA [27,28,29]. Therefore, the identification of unique miRNA-expression patterns in PALL may have great potential for clinical translational therapy.

We aimed to explore the role of PALL exosomes (Exo-PALL, derived from serum of children diagnosed with ALL or from conditioned medium (CM) of ALL cell lines) and its possible correlation to leukemic cell proliferation. Exo-PALL showed up-regulated miR-181a expression compared to exosomes-derived from healthy donors serum. Moreover, exosome-induced cell proliferation was reversible by silencing of Exo-miR-181a by a specific miR-181a inhibitor. Silencing of miRNA/mRNA at cellular level is widely accepted as an investigational tool, but miRNA-silencing at exosomal level is a rather new concept. Endo et al. demonstrated that cellular gene silencing by lipid nanoparticles, loaded with a gene-specific siRNA, significantly downregulated the expression of indoleamine 2, 3-dioxygenase 1. This study provides a proof-of-concept that microvesicles can be exploited as vehicles and/or delivery systems for siRNA [30].

## 2. Results

### 2.1. Characterization of Exosomes

**Figure 1:**
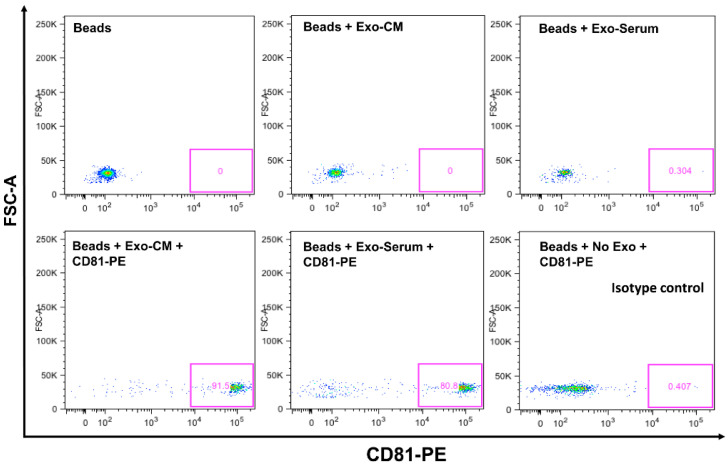
Expression of CD63 and CD81 on exosomes by flow cytometer. Binding/capture of exosomes was carried out with CD63 antibody coated magnetic beads overnight at room temperature. Bead captured exosomes were stained with primary antibody CD81 antibody (Biotin conjugated), then stained with secondary detection reagent (streptavidin-PE conjugated). Each condition was labeled for control purpose in the figure. Exo-serum represents, exosomes derived from serum. Exo-CM represents, exosomes derived from conditioned medium of JM1 cell.

Confirmation of CD63 and CD81 Expression on Exosomes by Flow Cytometer. CD63 and CD81 are expressed on exosomal surfaces and are well established as their biomarkers [31]. We explored CD63 and CD81 expression by flow cytometry on exosomes that were isolated by ultracentrifugation from serum and conditioned medium of leukemic cell lines. Analyzed data demonstrate that exosomes are positive for CD81 and CD63 expression (Figure 1). We have shown previously that exosomes are positive for CD63 and negative for calnexin by Western blotting [32]. We also performed nano-tracking analysis (NTA-Malvern) on exosomes and showed that 90% of the isolated exosomes are within the optimum size range of 50–100 nm diameter. Thus, by ultracentrifugation method, we are able to obtain the correct nano-vesicle population as identified by surface marker expression and size analysis [32].

### 2.2. Leukemia Derived Exosome-Induced Cell Proliferation

**Figure 2:**
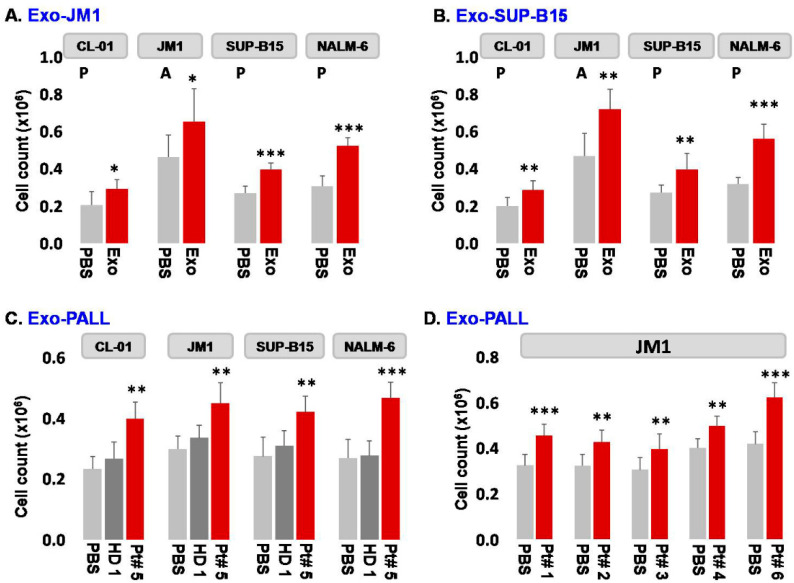
Exosomes from leukemia cell lines and from serum of pediatric ALL patients induce autocrine and paracrine cell proliferation in leukemia cell lines. (**A**) Exo-JM1-induced cell proliferation in control B cell line CL-01 and human leukemia cell lines JM1, SUP-B15, and NALM-6. (**B**) Exo-SUP-B15 - induced cell proliferation in control B cell line CL-01 and human leukemia cell lines JM1, SUP-B15, and NALM-6. (**C**) Exosomes derived from human serum of ALL patient #5 (Exo-PALL) at day 1 diagnosis induce proliferation in CL-01, JM1, SUP-B15, and NALM-6 cells compared to healthy donor exosomes (HD) or control PBS treated only. (**D**) Human serum exosomes (Exo-PALL) derived from the five different patients (Pt #1, 2, 3, 4 and 6) induce cell proliferation in JM1 cells compared to control PBS group. (PBS: control—no exosomes; P: paracrine effect; A: autocrine effect. Mean of 3 experiments. *p*-value * *p* < 0.05, ** *p* < 0.01, *** *p* < 0.001).

- Exosome isolation: Exosomes were isolated from conditioned media (Exo-CM) of two ALL cell lines (JM1 and SUP-B15) and one control B cell line (CL-01). In addition, exosomes were harvested from human serum samples of either patients with childhood acute lymphocytic leukemia (Exo-PALL) or healthy donors (Exo-HD).
- Exosome dose titration and exposure time optimization: In order to establish exosome dose titration and time kinetics of the process, Exo-CM (CL-01 and SUP-B15) was added to JM1 cells (paracrine incubation) in one of three different concentrations (100, 250, and 500 µg/mL) with PBS only (no exosomes) as negative control. Cell counting was carried out at 24 and 48 h under light microscopy. Control Exo-CL-01 did not induce cell proliferation at either time points, while Exo-SUP-B15 significantly induced cell proliferation, compared to PBS negative control, both at 24 and 48 h. Similar results were obtained with autologous incubation (autocrine fashion) of Exo-JM1 on JM1 cells (data not shown). Exosome treatment augmented cell proliferation at a starting exosome concentration (100 µg/mL), with optimal proliferation at 250 µg/mL (Supplementary Figure S1A). Consequently, we chose an exosome concentration of 250 µg/mL (noted saturated cell proliferation induction) as the working dose for future experiments. Similarly, we optimized the dose and time points from HD and PALL-derived serum exosomes. Further, JM1 leukemia cells were treated with Exo-HD and Exo-PALL. The control Exo-HD did not induce cell proliferation compared to PBS at either time points (24 or 48 h), while Exo-PALL significantly induced leukemic cell proliferation compared to Exo-HD and PBS negative control, both at 24 and 48 h (Supplementary Figure S1B). Based on these data, we decided that 250 µg/mL exosome concentration and 24 h incubation are the optimum conditions for the cell proliferation assay and we used these in the subsequent experiments.
- Exosome-induced leukemia cell proliferation: Consequently, we confirmed that CM-exosomes induce JM1 cell proliferation both in an autocrine and paracrine fashion using Exo-JM1 (Figure 2A) and Exo-SUP-B15 (Figure 2B), respectively. Our data showed that exosomes originated from ALL cell lines (JM1, SUP-B15) promoted cellular proliferation not only in both leukemic B cell lines but also in control human B cells (CL-01) as well (Figure 2A,B).

In addition, we wanted to explore the reproducibility of this cell proliferation effect with exosomes isolated from human serum from PALL patients (*n* = 6) (Figure 2C,D). Exo-PALL isolated from patient serum at day 1 of diagnosis (Patient #5) and Exo-HD from a healthy donor (HD #1) were incubated with control human B cells (CL-01) and leukemia cell lines (JM1, SUP-B15, and NALM-6). Only PALL serum-derived exosomes promoted cell proliferation in control human B cells (CL-01) as well as in leukemic B cell lines (JM1, SUP-B15, and NALM-6) (Figure 2C). To consolidate our data, JM1 cells were treated with Exo-PALL from five different patients (Pt #1, 2, 3, 4, and 6). All five Exo-PALL enabled significant induction of cell proliferation in JM1 cells (Figure 2D). Apart from cell counting using trypan blue, we also confirmed our cell proliferation experiment by MTS assay (Supplementary Figure S2), supporting that leukemia-derived exosomes induce augmented cell proliferation.

### 2.3. Exo-JM1 and Exo-SUP-B15 Treatment Augments Proliferative/Pro-Survival Genes, and Down Regulates Pro-Apoptotic Genes

**Figure 3:**
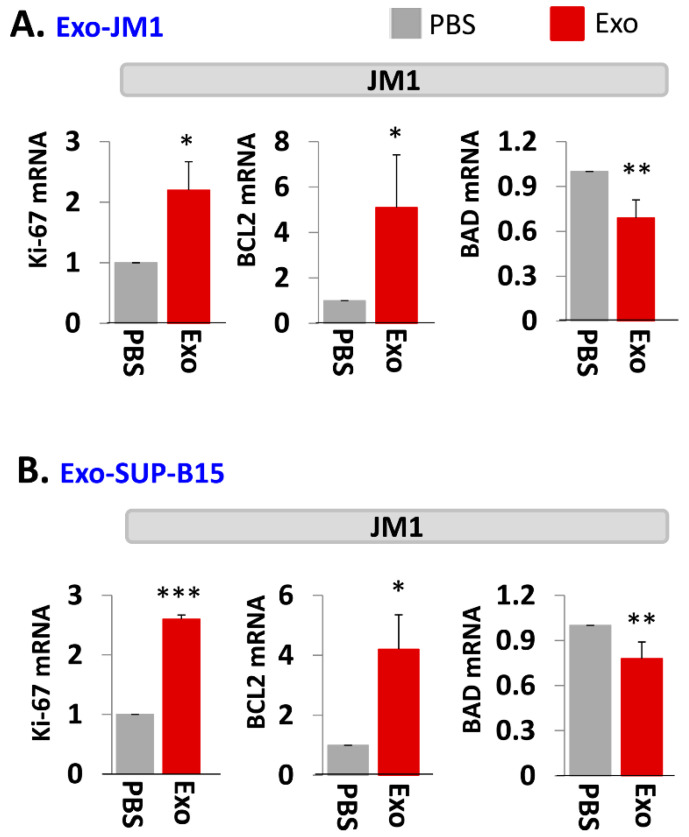
Exo-JM1 and Exo-SUP-B15 regulate proliferative, pro-survival, and pro-apoptotic genes in JM1 cells. (**A**) Exo-JM1 up-regulates Ki-67, and BCL2 mRNA expression (pro-survival) and down-regulates BAD mRNA expression (pro-apoptotic) in autocrine manner. (**B**) Exo-SUP-B15 promotes Ki-67 and BCL2 mRNA expression and down regulates BAD expression in JM1 cells in paracrine manner. Data represented are mean of three experiments. (Ctrl: PBS only—no exosomes. *p*-value * *p* < 0.05, ** *p* < 0.01, *** *p* < 0.001).

In order to evaluate one of the molecular pathways by which Exo-JM1 and Exo-SUP-B15 promotes cell survival and cell proliferation, we analyzed gene expression profiles in the JM1 leukemia cells after exosomes exposure. Both Exo-JM1 and Exo-SUP-B15 altered the mRNA expression of regulatory genes such as Ki-67, BCL2, BAD in JM1 cells (Figure 3A,B). Exo-CM from ALL cell lines (JM1 and SUP-B15) were added to JM1 cells and proliferative (Ki-67, PCNA), pro-survival (MCL1, BCL2) and pro-apoptotic (BAD, BAX) genes were analyzed by mRNA expression at two time points (6 and 24 h). For both time points, incubation of JM1 cells with Exo-JM1 (autocrine exposure) resulted in an up-regulated expression of the proliferative/pro-survival genes Ki-67, PCNA, MCL1, and BCL2, while it down-regulated the expression of the pro-apoptotic genes BAD and BAX (Supplementary Figure S3A). To authenticate these changes in gene expression in cells that were exposed to leukemic exosomes, we also tested Exo-SUP-B15 on JM1 cells (paracrine exposure). We found a similar pattern of augmented expression of Ki-67, PCNA, MCL1, and BCL2 mRNA and down-regulated expression of BAD and BAX mRNA at both 6 and 24 h induced by Exo-SUP-B15 (Supplementary Figure S3B). Moving forward, we selected an incubation time of 24 h to replicate the results in other experiments. Overall, our current data suggest that both Exo-JM1 and Exo-SUP-B15 promote cell proliferation by enhancing proliferative/pro-survival signals and suppressing pro-apoptotic genes.

### 2.4. Exo-PALL Promotes Proliferative/Pro-Survival Genes, and Down-Regulates Pro-Apoptotic Genes

**Figure 4:**
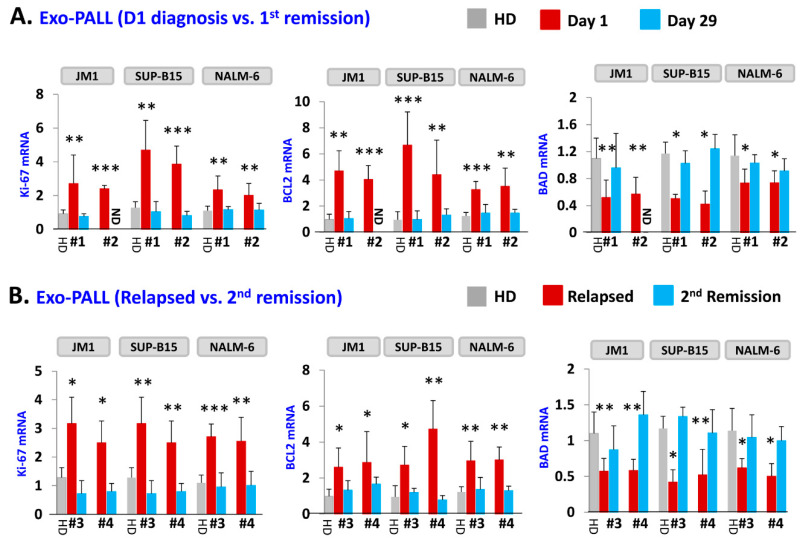
Exo-PALL treatment contributes to gene regulation in leukemia cell lines which correlates with disease stage. Three different cell lines were exposed with indicated Exo-PALL isolated from serum of Pt #1 (Day1 diagnosis), Pt #2 (Day29 1st remission), Pt #3 (first relapse) and Pt #4 (2nd remission). The expression of mRNA was evaluated by q-PCR. (**A**) Exo-PALL Pt #1 up-regulates Ki-67 and BCL2 mRNA expression and down-regulate BAD mRNA expression in JM1, SUP-B15 and NALM-6 leukemia cell lines compared to HD exosomes. This augmentation effect of Exo-PALL on mRNA expression is no longer detectable during remission (Pt #2; Day 29-remission). (**B**) Similar effect is obtained with Exo-PALL Pt #3 and #4 at first relapse and in 2nd remission. (Pt #1, #2, #3 and #4: Exo-serum samples of four different patients. HD: healthy donor control serum). (*p*-value * *p* < 0.05, ** *p* < 0.01, *** *p* < 0.001).

Next, we tested the effect of exosomes of serum samples from PALL patients and healthy donors on gene induction (Figure 4). Three leukemia cell lines (JM1, SUP-B15, and NALM-6) were co-cultured with exosomes. To confirm the results obtained from Exo-CM, we explored if Exo-PALL induced the same changes in gene expression involved in the proliferation, survival and apoptotic cellular pathways. We used exosomes from serum of patients at different stages of leukemia (at diagnosis, first remission, relapse and second remission). Exo-PALL at the diagnosis (Day 1) augmented Ki-67 and BCL2 while BAD gene expression appeared to be down-regulated (Figure 4A). Interestingly, Exo-PALL from Day 29 (1st remission) failed to induce Ki-67 and BCL2 in target cells, while the BAD gene was up regulated (Figure 4A). This experiment was carried out in three different target leukemia cell lines. We also compared the potential of human serum exosomes isolated from PALL relapse versus PALL 2nd remission (Pt #3 and Pt #4). PALL relapse exosomes exposure up-regulated Ki-67, BCL2 and down regulated BAD mRNA expression (Figure 4B). In contrast, incubation of target cells with PALL 2nd remission-derived exosomes failed to up-regulate Ki-67 and BCL2 mRNA and failed to down-regulate BAD mRNA expression (Figure 4B). These experiments were also carried out in three different target leukemia cell lines with consistent results obtained.

### 2.5. Analysis of miR Profiles in Exosomes

#### Exo-miR Expression by Human Cancer Pathway Finder Array and Validation of Elevated Exosomal miR-181a Expression by q-PCR

**Figure 5:**
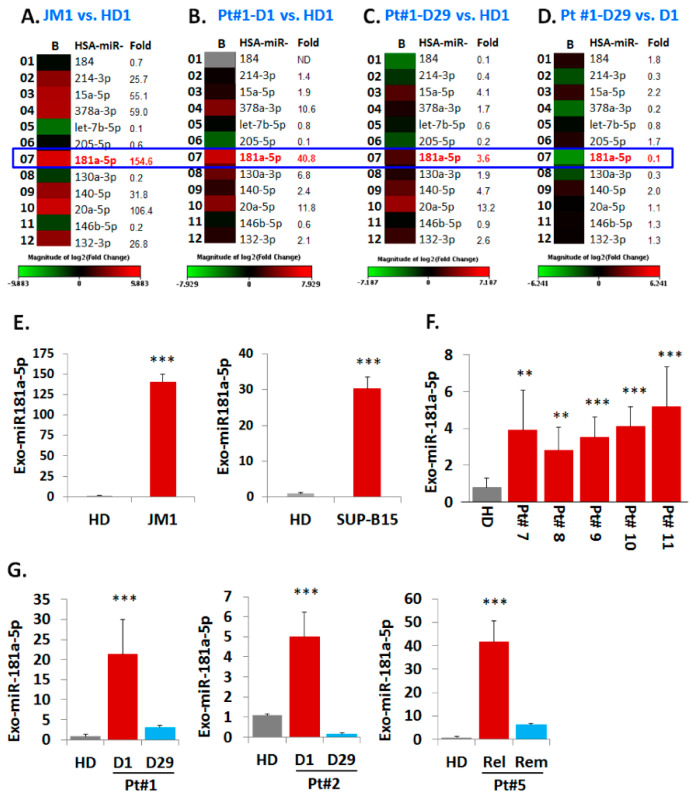
Heat map analysis of Exo-miR array by human cancer pathway finder and validation of exosomal miR-181a expression by q-PCR. Representative example of a heat map analysis shows differential miRNA expression between two groups overlaid onto PCR Human Cancer Pathway Finder Array plates show. (**A**) miR-181a is 154fold up-regulated in exosomes of JM1 cell line compared to HD exosomes; (**B**) miR-181a is 40 fold up-regulated in Exo-PALL of Pt #1 at diagnosis (D1) compared to HD. (**C**) miR-181a is only 3.6 fold high in the same Exo-PALL (Pt #1) in remission (D29) compared to HD. (**D**) miR-181a is at baseline in Exo-PALL (Pt #1) upon remission (D29) compared to diagnosis (D1). (**E**) Expression of miR-181a-5p by q-PCR in Exo-CM of ALL cell lines JM1, and SUP-B15 compared to HD. (**F**) Exo-miR-181a expression in five PALL patients’ (Pt #7, 8, 9, 10 and 11) samples by q-PCR compared to HD. (**G**) Expression of miR-181a-5p in Exo-PALL patients at diagnosis (D1), remission (D29), first relapse (relapse) and 2nd remission (remission) in three different ALL patient samples (Pt #1, Pt #2, & Pt #5). Data represented are mean of triplicates. (*p*-value ** *p* < 0.01, *** *p* < 0.001).

Exosomes from both CM of cell lines and serum samples (PALL and HD) were analyzed for their miRNAs expression profiles (Figure 5). We chose the Human Cancer Pathway Finder Array (Qiagen) which allowed us to screen for 84 different miRNA commonly found in cancer. We found that exo-miR-181a-5p expression was 154-fold higher in JM1 cells (Figure 5A). Exo-miR-181a-5p was 40-fold higher in exosomes of PALL D1 (at day 1 of diagnosis, Pt #1) (Figure 5B) but its expression reverted back to almost normal once the patient achieved remission at Day 29 (PALL D29 Pt #1) (Figure 5C). When comparing exo-PALL D29 vs. exo-PALL D1 in samples derived from the same patient (Pt #1), miR-181a-5p expression was 11-fold down regulated in the Day 29 (remission) samples compared to D1 (diagnosis) (Figure 5D). We validated exo-miR-181a-5p expression in ALL cell lines and multiple PALL patients by q-PCR. The miR-181a expression in Exo-JM1 was 140-fold higher and in Exo-SUP-B15 was 30-fold higher relative to control HD serum exosomes (Figure 5E). Exo-miR-181a-5p was also screened in five different PALL patients and data showed 3–5-fold higher expression of miR-181a-5p (Figure 5F). In serum exosomes of PALL patient #1, miR-181a-5p level at D1 of diagnosis was 20-fold higher compared to a healthy donor, while at D29 (1st remission) it was only three-fold higher compared to the control (Figure 5G 1st graph). Similarly, in PALL Pt #2, miR-181a-5p expression in serum exosomes at D1 of diagnosis was higher (five-fold) than in HD, while at D29 (remission) decreased to 0.15-fold compared to the control (Figure 5G 2nd graph). When comparing relapse vs. second remission, serum exosome miR-181a-5p expression in PALL Pt #5 was 42-fold higher during relapse compared HD and decreased during 2nd remission, although still remained 6-fold higher than the healthy control (Figure 5G 3rd graph). Taken together, our data indicate that PALL D1 or relapse-derived exosomes are loaded with miR-181a-5p at the time of active disease status. Based on this, we hypothesized that leukemic exosomal miR-181a-5p could play a role in leukemia cell proliferation.

### 2.6. Transfection Efficiency of TexRed-siRNA into the Target Cell and Silencing of exosomal miR-181a

**Figure 6:**
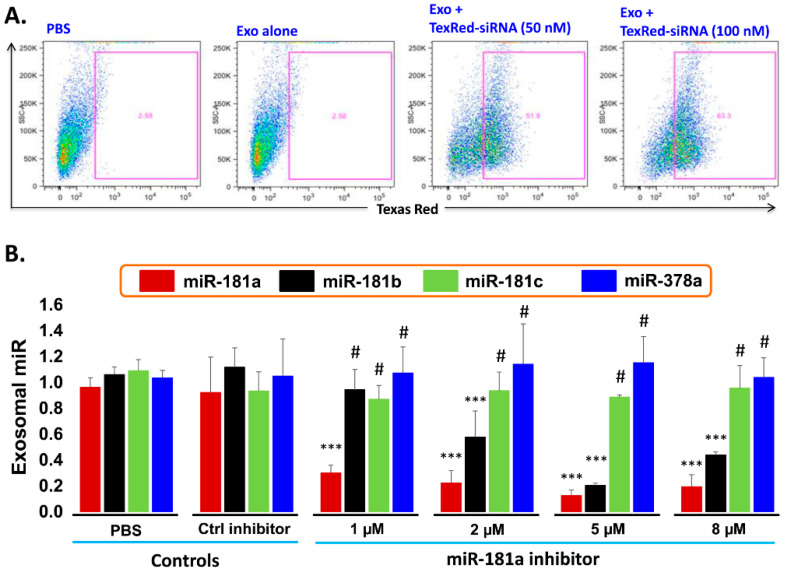
Cellular uptake efficiency of TexRed-siRNA and dose optimization of miR-181a-5p inhibitor. (**A**) TaxRed-siRNA transfection efficiency into Exo-JM1 and cellular uptake of exosome was measured by flow cytometer. FACS data show that 50–60% JM1 cells are TexRed positive compared with PBS/Exo alone control group. (**B**) Exosome transfection with Exo-miR-181a-5p inhibitor for miR-181a-5p silencing. Exo-JM1 were transfected with 1, 2, 5, 8 µM of miR-181a inhibitor. Exo-miR-181a-5p expression was carried out by q-PCR. Transfection of exosomes with an inhibitor resulted 70-88% silencing of exosomal miR-181a. An unrelated miR-181b, miR-181c, and miR-378 was amplified from the same cDNA by q-PCR. SNORD61 was used as endogenous miR. (*p*-value *** *p* < 0.001. #: not significant). Data was analyzed between control groups vs. miR-181a inhibitor treated group.

- Transfection efficiency of siRNA inhibitor in exosomes: To support the idea that miR-181a is a major player in leukemia cell proliferation, we explored miR-181a inhibition by a miScript miRNA inhibitor to reverse the induced cell proliferation. We first established and determined uptake-efficiency of a control siRNA by exosomes, by TexRed as per manufacturer recommendations (Figure 6). We transfected and loaded Exo-JM1 with TexRed-siRNA and co-cultured with JM1 cells. After 24 h, JM1 cells were harvested and analyzed for TexRed-siRNA uptake by the flow cytometer. We observed around 50–60% JM1 cells were TexRed positive (Figure 6A).
- Targeted silencing of miR-181a by siRNA inhibitor: Once siRNA uptake was confirmed, Exo-JM1 was transfected and loaded with a specific miR-181a inhibitor and the exosomal miR-181a level was determined by q-PCR. Results showed that transfection of exosomes with an inhibitor resulted in a 70–88% silencing of exosomal miR-181a (Figure 6B). To rule out off-target activity causing non-specific inhibition, we randomly chose to amplify miR-181b, miR-181c, and miR-378 as an off-target hits. The miR-181a inhibitor consistently inhibited specific miR-181a expression at all applied concentrations (1–8 µM). The miR-181a inhibitor did not inhibit non-specific miR-181b at low concentration (1 µM) while miR-181a inhibitor at high concentration (2–8 µM) showed off target activity and miR-181b inhibition was observed. Interestingly, both miR-181c and miR-378a was not inhibited by miR-181a inhibitor at either concentration.

### 2.7. PALL-Derived Exosomal miR-181a Silencing by miR-181a Inhibitor Reverses Exosome Induced Cell Proliferation

**Figure 7:**
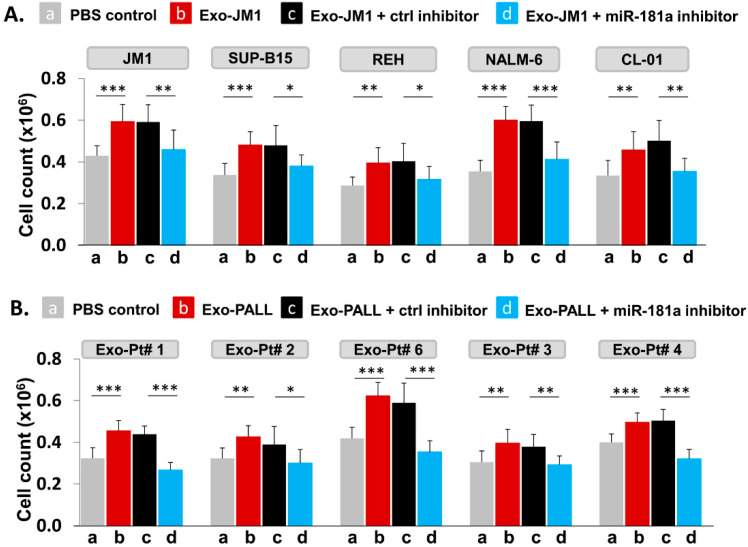
Leukemia derived-exosomal miR-181a silencing reverses exosome-induced cell proliferation. (**A**) Silencing of miR-181a in Exo-JM1 (CM) impairs cell proliferation: Indicated cells were treated with (a) PBS, (b) Exo-JM1, (c) Exo-JM1 + control inhibitor, (d) Exo-JM1 + miR-181a inhibitor (1.0 µM) for cell proliferation assay. Cell proliferation was observed after treatment with wild type Exo-JM1 (b) or with Exo-JM1+ control inhibitor (c) while in the Exo-JM1 + miR-181a inhibitor treated group (d), cell proliferation remained at baseline, similar to PBS-treated cells (a). These experiments were repeated in five different cell lines: JM1, SUP-B15, REH, NALM-6, and CL-01 as indicated. (**B**) Silencing of miR-181a in Exo-PALL (serum) impairs cell proliferation: JM1 cells were treated with (a) PBS, (b) Exo-PALL, (c) Exo-PALL + control inhibitor, (d) Exo-PALL + miR-181a inhibitor in cell proliferation assay. Cell proliferation was observed after treatment with Exo-PALL (b) or with Exo-PALL+ control inhibitor (c) while in the Exo-PALL + miR-181a inhibitor treated group (d), cell proliferation remained at baseline, similar to PBS-treated cells (a). Exo-PALL was derived from five different P-ALL patient samples (Pt #1, 2, 3, 4 and 6). (*p*-value * *p* < 0.05, ** *p* < 0.01, *** *p* < 0.001).

We hypothesized that PALL exosome-induced proliferation could be reversed by silencing of Exo-miR-181a by a specific miR-181a inhibitor (Figure 7). To explore further the role of miR-181a in cell proliferation, we treated leukemia cell lines for 24 h with Exo-JM1 that were prior transfected with a miR-181a inhibitor in following conditions: PBS control (a), Exo-JM1 alone (b), Exo-JM1 + ctrl inhibitor (c), and Exo-JM1 + miR-181a inhibitor (d). Induced cell proliferation was observed after treatment with Exo-JM1 alone (b) or with Exo-JM1 + control inhibitor (c), while in the Exo-JM1 + miR-181a inhibitor treated group (d), cell proliferation remained at baseline, similar to PBS-treated cells (a). We repeated these observations in five different cell lines (JM1, SUP-B15, REH, NALM6, and CL-01). All five cell lines showed a reproducible similar pattern of exosome-induced cell proliferation and this effect was reversed in the presence of the exosomal miR-181a-5p inhibitor (Figure 7A). These results suggest that miR-181a is functionally active in leukemia exosomes, contributing to the induction of cell proliferation.

To confirm proof of concept, we treated JM1 cells with exosomes derived from five different PALL patient samples with or without prior miR-181a silencing by an inhibitor in the cell proliferation assay (Figure 7B). We observed, Exo-PALL alone (b) and Exo-PALL + ctrl inhibitor (c) was able to induce cell proliferation compared to PBS control (a) while Exo-PALL + miR-181a inhibitor treated group (d) could not induce cell proliferation (Figure 7B). We obtained consistent outcomes in all five Exo-PALL samples. These results suggest that Exo-miR-181a from PALL patients’ serum contribute to leukemia cell proliferation, similarly to the results obtained from CM exosomes. Overall, our data suggest that exosomal miR-181a is a biologically active player with a functional role in leukemia cells that leads to induced cell proliferation.

## 3. Discussion

In this study, we identified that exosomes induce cell proliferation in ALL B-lymphocytes as well as in control B-lymphocytes. Furthermore, we showed that cell proliferation is promoted by the up-regulation of exosomal-miR-181a expression and reversible by mi-R181a silencing. The literature illuminates that exosome dose optimization for functional studies varies from cell type to cell type. For example, in gastric cancer, an exosomal dose of 50–400 µg/mL shows a dose-dependent cell proliferation [23]. Exosomes derived from mesenchymal cells showed different dose & time-dependent angiogenesis in breast cancer [33]. We titrated and optimized the dose of PALL exosomes for optimal levels of induction of cell proliferation. We show that, at both 100 µg/mL and 250 µg/mL exosome dosing, cell proliferation was induced dose-dependently, but at higher doses, we observed a plateau effect. We decided that for our studies with leukemia cell lines, a working concentration of 250 µg/mL was optimal.

Exosomes derived from conditioned media (CM) of ALL cell lines and PALL patient’s serum are capable to induce leukemic cell line proliferation, and this process seems highly specific and not at random. Analysis of proliferative/pro-survival genes and pro-apoptotic genes revealed the upregulation of the former and down-regulation of the latter after exosome exposure. Similarly, others have shown that chronic myeloid leukemia (CML) cell line LAMA84-derived exosomes promote proliferation genes (BCL-xl, BCL-w, survivin) and downregulate pro-apoptotic genes such as BAD, BAX, and PUMA [34]. However, there are no reports in the literature on acute lymphocytic leukemia-derived exosomes cell proliferation by altered gene expression profiles.

To explore the physiological mechanism behind the above results, we screened 84 different exo-miRs by use of the Human Cancer Pathway finder PCR array. We found that miR-181a was uniquely and significantly amplified in exosomes derived from P-ALL serum and leukemia cell lines. Furthermore, we showed that inhibition of exosomal miR-181a curbed the exosome induced cell proliferation. Based on our study, we suggest that exo-miR-181a might play a significant part in leukemic cell proliferation. While reports exist on miR-181a and cell proliferation, this is the first report delineating the effect of ALL-derived exosomes and exosomal miR-181a on cell proliferation. Signaling of miR-181a involves target genes of Wnt-signaling which lead to cell proliferation in ALL by inhibiting WIFI signaling [35]. Verduci et al. reported that elevated miR-181a enhances cell proliferation in acute lymphoblastic leukemia by targeting EGR1 [36]. In solid tumors, miR-181a has been described to play an important role in cancer progression, i.e., augmented miR-181a expression promotes prostate cancer cell proliferation by down-regulating DAX-1 expression and up-regulating PSA, CDK1, and CDK2 [37]. Over-expression of miR-181a leads to increased viability of osteosarcoma cells and induced cell proliferation by augmenting BCL2, MMP9; and apoptosis was inhibited by down-regulation of p21 and TIMP3 expression [38]. Moreover, enhanced expression of miR-181a promotes cell proliferation and inhibits apoptosis in gastric cancer by targeting MTMR340, via RASSF6-MAPK signaling activation [39].

Our novel data shows that the silencing of miR-181a in leukemia-derived exosomes reverses their effect on leukemic cell proliferation. This suggests that leukemia-derived exosomes are functional and capable to transfer their cargo via exosomal cellular uptake to interfere with the cellular programming of the target cell.The silencing of miR-181a specifically at the exosomal level was challenging. We optimized exosome transfection efficiency and cellular uptake of exosomal contents into the target cells. First, we established that exosome uptake efficiency into the target cells is ~50–65% for Texas-Red conjugated-siRNA, similar to what others have reported for TexRed labeled exosomes in HuH-7 cells [40], and alexa-flour-488-conjugated siRNA labeled exosomes into the HTB-177 cells [41]. Exosome uptake and cargo delivery into target cells involve multiple mechanisms, such as soluble signaling, juxtacrine signaling, membrane fusion, phagocytosis, macro-pinocytosis, and receptor/raft mediated endocytosis [42]. Besides, we showed that successful transfection with a miR-181a inhibitor into exosomes resulted in ~ 80–90% inhibition of exo-miR-181a expression. The miR-181a inhibitor was highly specific to miR-181a since it did not silence the expression of another randomly selected miR, namely miR-378a, and miR-181c. Interestingly, expression of miR-181b was inhibited at high concentration of miR-181a inhibitor (2–8 µM) while no inhibition of miR-181b was observed at low concentration of miR-181a inhibitor (1 µM). We used 1.0 µM miR-181a inhibitor in our study. Other groups have attempted exosomal silencing by siRNA with similar results; i.e., exosomal delivery of a PLK-1 gene-specific siRNA silencer in UMUC3 bladder cancer cell lines showed >60% silencing of PLK-1 expression [43]. Endo et al. used a lipid nanoparticle as delivery system carrying siRNA (90% encapsulation of siRNA into the lipid nanoparticle) for the silencing of indoleamine2, 3 dioxygenase 1 gene which has implications for cancer immunotherapy [30]. However, exosome-based siRNA-inhibitor delivery can be considered a better vehicle system than liposomal-based or other vector delivery systems for several reasons: (1) Exosomes are biological nano-carrier systems that easily communicate with target cells for exosomal cargo delivery. (2) Exosomes harvested from the patient’s own body fluids or cell culture conditioned medium are non-immunogenic and can be utilized for autologous loading of a biological agent. (3) Exosomes are considered non-toxic in contrast to other transfection reagents.

Our data support that miR-181a silencing in exosomes reverses exosome-induced cell proliferation. Although some reports in the literature indicate that cellular inhibition of miR-181a suppresses cell growth and proliferation [35,39], none have reported the effect of exosomal miR-181a silencing upon cell proliferation in ALL. Exosomal silencing of targeted miRs involved in cancer progression might allow for new venues to develop cancer therapeutics.

## 4. Materials and Methods

### 4.1. Cell Lines and Cell Culture

ALL cell lines were purchased from ATCC (SUP-B15, JM1 and Nalm-6/CRL-1929™, CRL-10423™ and CRL-3273™) and REH cells from DSMZ (Cat# ACC 22). CL-01 was donated by the Chiorazzi lab, Feinstein Institutes for Medical Research, Manhasset, NY. Exosomes were isolated by ultracentrifugation from the CM of cell lines (Exo-CM) as described [44]. The miRNA silencing inhibitors were obtained from Qiagen as follows: miScript inhibitor negative control (20 nmol, Qiagen, cat# 1027272), anti-hsa-miR-181a-5p miScript miRNA Inhibitor (20 nmol, Qiagen, cat# MIN0000256).

### 4.2. Human Serum Samples

All subjects gave their informed consent for inclusion before they participated in the study. The study was conducted in accordance with the Declaration of Helsinki, and the protocol was approved by the Ethics Committee of Northwell Health Institutional Review Board (IRB# HS16-0253, date of initial IRB approval 4/12/2016). PALL patients (*n* = 11) and healthy donor (HD) (*n* = 4) serum samples were used in this study (Supplementary Table S1). P-ALL serum samples were collected at different leukemia disease stages: new diagnosis (Day 1), remission (Day29), relapse or second remission. Exosomes were isolated from serum by ultracentrifugation (Exo-PALL) [44].

### 4.3. Depletion of Exosomes from the FBS

To avoid contamination by exogenous exosomes contained in the fetal bovine serum (FBS) that is added to culture medium, FBS was converted into exosome-free FBS by ultracentrifugation [44]. Briefly, FBS was subjected to centrifugation for 10 min at 300× *g*. Supernatant was collected and centrifuged for another 10 min at 2000× *g*. Again, supernatant was collected and centrifuged for 30 min at 10,000× *g*. Then, supernatant was ultra-centrifuged overnight at 100,000× *g*. The following day, FBS supernatant was collected and labeled as exo-free FBS. Each step was carried out at 4 °C.

### 4.4. Cell Culture Set up for Exosome Production

Each leukemic producer cell line was cultured in exo-free FBS cell culture medium during cell expansion in order to harvest exosomes of conditioned medium (CM). Cells were plated (1 × 10^6^/mL) in 10 mL total volume in a 100-mm tissue culture dish. After 48 h, CM of each cell line was harvested by centrifugation and filtered through a 0.22 micron filter (Millipore) to remove cell debris. Filtered CM was used for exosome isolation by ultracentrifugation.

### 4.5. Exosome Isolation

Exosomes were purified by ultracentrifugation method as described [44]. Briefly, human serum and CM from cell lines were subjected to centrifugation for 10min at 300× *g*. Supernatant was collected and centrifuged for 10 min at 2000× *g*. Again, supernatant was collected and centrifuged for 30 min at 10,000× *g*. Then supernatant was ultra-centrifuged for 120 min at 100,000× *g*. Pellets were re-suspended in PBS and again ultra-centrifuged for 120 min at 100,000× *g*. Pellets containing exosomes were harvested and reconstituted in 250 μL PBS. Each centrifugation step was carried out at 4 °C. The protein content of the exosomes was measured using a BCA protein assay kit (Bio-Rad). Exosomes were aliquoted and stored at −80 °C for further usage.

### 4.6. Characterization of Exosomes

#### Exosomal CD63 and CD81 Expression by Flow Cytometer

We used the exosome isolation and analysis kit (cat: ab239682) from Abcam as per manufacturer protocol. Briefly, exosomes (10 µg) were taken into 50 µL PBS. Captured beads (100 µL) were mixed with 50.0 µL of exosomes in a flow cytometer tube. The mixture was incubated at room temperature in the dark overnight. The primary detection antibody (CD81-biotin-conjugated) (5.0 µL) was added and incubated at 2–8 °C for 1.0 h in the dark. Washing steps: 1.0 mL of assay buffer was added to samples, mixed by tapping, and centrifuged at 2000 rpm for 5.0 min. The supernatant was decanted and pellets were reconstituted in 100 µL assay buffer. A secondary antibody (streptavidin-PE conjugated) (5.0 µL) was added to each tube and incubated at 2–8 °C for 30 min in the dark. Then, the washing step was repeated. Exosome pellets were reconstituted in 350 µL assay buffer and read at flow cytometer (Fortessa).

### 4.7. Exosome Induced-Cell Proliferation Assay

JM1, SUP-B15, and NALM-6 leukemia cell lines (0.2 × 10^6^/well) were seeded in 96-well plates and cultured in exo-free FBS medium. We also tested primary exosomes purified from HD and four P-ALL patients at different disease stage: P-ALL D1 (day 1 of diagnosis), and P-ALL D29 (1st remission) (pt #1 and pt #2), P-ALL relapse, P-ALL 2nd remission (pt #3 and pt #4) in three leukemia cell lines. Each well was loaded with exosomes (250 µg/mL) and incubated at 37 °C. After 24 h, cells were mixed by gently pipetting. Cell proliferation was analyzed by live-cell counting using the trypan blue/hemocytometer method under the microscope.

### 4.8. Cellular RNA Extraction and cDNA Preparation

Total RNA was extracted from SUP-B15, JM1, or CL-01 cells by Trizol reagent (Invitrogen) method. The quality and quantity of RNA were analyzed by the NanoDrop ND1000 spectrophotometer. Around 2–5 µg of total RNA was used for cDNA synthesis by q-PCR. Oligo-dT primers (Invitrogen) and M-MLV reverse transcriptase (cat # 28025-013, Invitrogen) were used for cDNA synthesis as per manufacturer’s protocol.

### 4.9. Cellular mRNA Expression by q-PCR

Primer sequences, probe numbers, and gene accession numbers from the universal probe library (UPL) of Roche Applied Science are described (Supplementary Table S2). Gene amplification was carried out by q-PCR with cDNA as template and Eurogentec master mix. Fold change was calculated by comparing treated vs. untreated groups. Data were analyzed with RQ manager version 1.2.1 (Applied Biosystems, Foster City, CA, USA). The data are expressed as fold change. GAPDH was included as an endogenous reference gene. Exo-JM1 and Exo-SUP-B15 were loaded on JM1 cells. After 24 h of exosome co-culture, cells were harvested and washed. The mRNA was extracted for q-PCR analysis for the proliferative (Ki-67, PCNA), pro-survival (MCL1 and BCL2) genes; and BAD and BAX for pro-apoptotic pathways.

### 4.10. Exosomal RNA Isolation

CM was harvested from JM1 and SUP-B15 (2 × 10^6^ cells/mL) leukemic producer cell lines after 48 h of tissue culture and exosomes were isolated from CM by ultracentrifugation (10 min at 300× *g*, 10 min at 2000× *g*, and 30 min at 10,000× *g*). Collected CM was processed for isolation of exosomal RNA (ExoRNeasy Kit (MAXI)-Qiagen). Similarly, exosomal RNA from human serum samples was extracted utilizing the ExoRNeasy Kit (MIDI) (cat # 77044). RNA was quantitated by NanoDrop.

### 4.11. Silencing of Exosomal miR-181a with miR-181a Inhibitor Using Exo-Fect™ Exosome Transfection Reagent Kit

Exosomes were transfected with miR-181a inhibitor using Exo-Fect™ Exosome Transfection Reagent kit (cat # EXFT20A-1, SBI System Biosciences) according to manufactures protocol. Briefly, reaction mixture (150 µL) were prepared in 1.5 mL sterile plastic tube: Exosomes (50–300 µg) in (50 µL 1× PBS) + Exo-Fect solution (10.0 µL) + miR-181a inhibitor (1–8 µM) (20 µL) + 1× PBS (70 µL). Total transfection reaction volume (150.0 µL) were mixed well by flicking/inversion three times and was not vortexed. The reaction mixture was incubated at 37 °C in a shaker for 10 min and then immediately place the tube on ice. Reactions were stopped by adding ExoQuick-TC reagent (30 µL) provided in the kit and mixed by inverting 6 times. Do not vortex. Transfected exosome samples were placed on ice (or at 4 °C) for 30 min. After that, samples were centrifuged for 3 min at 13,000–14,000 rpm and the supernatant was removed. Then, pellets/transfected exosomes were re-suspended in 300 µL 1× PBS.

### 4.12. Exosomal miR-181a-5p Expression by PCR

Exo-RNA was converted into cDNA by miScript II RT kit (Cat # 218161, Qiagen). Expression of miRNA was evaluated by q-PCR using a miScript SYBR@Green PCR kit (Cat # 218073, Qiagen). Screening of miRNA expression for 84 different miRNAs was performed on Exo-RNA isolated of CM cell lines and human serum exosomes, using a Human Cancer Pathway Finder miScript miRNA PCR Array (Cat # MIHS-102ZF, 331221-Qiagen). Validation of miRNA was carried out by q-PCR using primers (miR-181a: cat # MS00008827, miR-181b: cat# MS00006699, miR-181c: cat# MS00008841 miR-378a: cat# MS0000690) from Qiagen.

### 4.13. Exosomal miR-181a Silencing Using a Specific miR-181a Inhibitor

- Confirmation of exosomal uptake of TexRed-siRNA and cellular uptake of exosomes: Exosomes were labeled with TexRed-siRNA (SBI System Bioscience) as per manufacturer’s protocol. TexRed-labeled JM1 exosomes were then co-cultured with JM1 cells for 24 h. Presence of TexRed-siRNA (introduced in target cells by exosomes) into the JM1 cells was assessed by flow cytometer.
- Dose titration of miR-181a inhibitor: Exo-JM1 were transfected with different concentrations of the miR181a inhibitor (1, 2, 5, 8 µM) to determine optimal dosing. Then, RNA was isolated from transfected exosomes for cDNA synthesis and miR-181a expression by q-PCR.
- Exosomal miR-181a silencing: 300 µg of exosomes (either CM- or serum-derived) was transfected under following conditions: (1) transfection reagents only; (2) transfection reagent and control inhibitor; (3) transfection reagent with miR-181a inhibitor (1.0 µM). Each transfection mixture was co-cultured with JM1 cells for 24 h, allowing for exosomal uptake by the target cells. Cell count was carried out by light microscopy.

### 4.14. Statistical Analysis

To compare the mean values between two groups, the unpaired t-test was used. Statistical significance was defined as *p* < 0.05. All results are represented as Mean ± SD. Each experiment was performed in triplicates.

## 5. Conclusions

Exosomal miR-181a expression is abundantly up-regulated in exosomes derived from P-ALL patients’ serum and CM of ALL cell lines and correlates with leukemia cell proliferation. Subsequent silencing of Exo-miR-181a reverses exosome-induced leukemia cell proliferation. Therefore, exosomal miR-181a inhibition can act as a novel synergistic pharmaceutical target for growth-suppression in pediatric acute lymphocytic leukemia.

## Supplementary Materials

The following are available online at https://www.mdpi.com/1424-8247/13/9/241/s1, Figure S1: Dose titration and time kinetics of exosomes enhancing cell proliferation. Figure S2: Exosome-induced cell proliferation by MTS assay. Figure S3: Exo-CM regulates proliferative, pro-survival, and pro-apoptotic genes. Table S1: List of healthy donors and PALL serum samples. Table S2: Human primers from universal probe library (UPL).
