# Supplementary material for: Silencing of Exosomal miR-181a Reverses Pediatric Acute Lymphocytic Leukemia Cell Proliferation
Source: Pharmaceuticals (Basel). 2020 Sep 11;13(9):241. doi: 10.3390/ph13090241 (PMC7558769; doi:10.3390/ph13090241)
Supplement: Supplementary file 1 [file pharmaceuticals-13-00241-s001.pdf]

pharmaceuticals

Supplementary

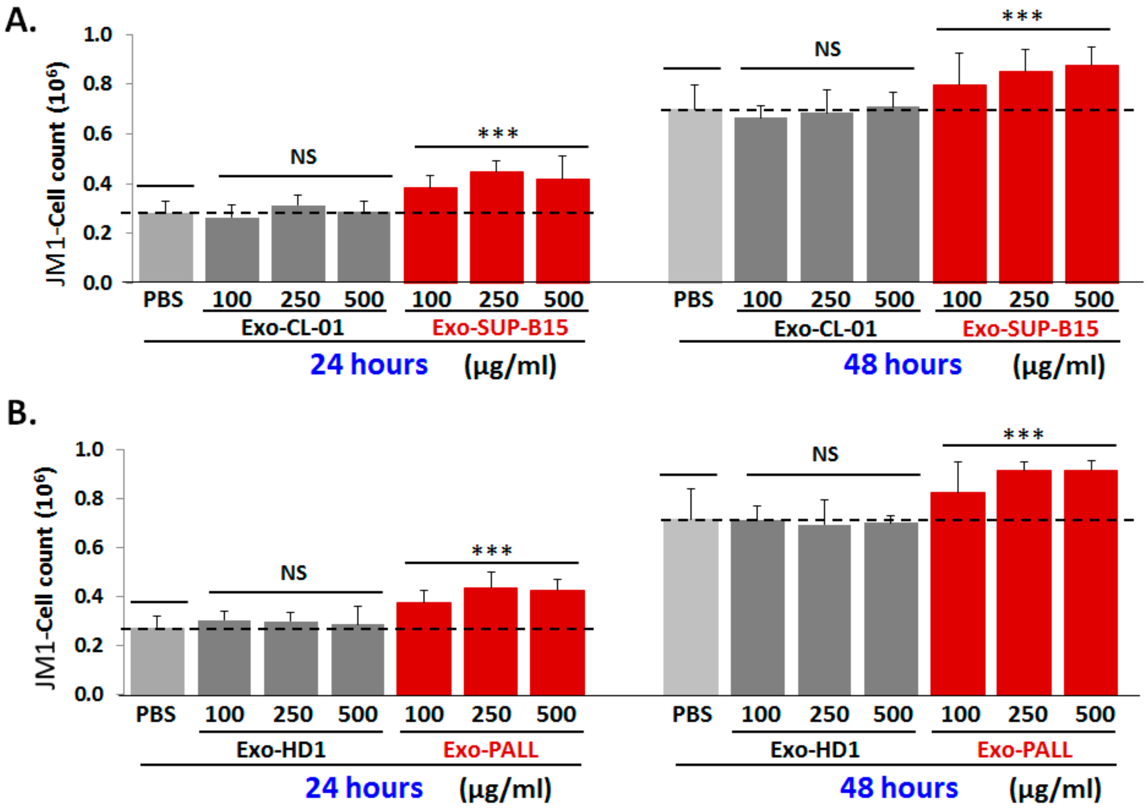

Figure S1. Dose titration and time kinetics of exosomes enhancing cell proliferation. (A) JM1 cells were treated with Exo-CL-01 (normal B cell line) and Exo-SUP-B15 (leukemia B cell line) in three different dosages (100, 250, 500 µg/ml). Cells were counted at 24 hours and 48 hours after treatment. **(B)** JM1 cells were loaded with Exo-HD and Exo-PALL in three different dosages (100, 250, 500 µg/ml) in JM1 cells. Cells were counted at 24 hours and 48 hours after treatment. Exosomes induced cell proliferation was present at optimal concentration of 250 g/ml of exosomes. *(P value * p<0.05, **p<0.01, ***p<0.001. NS; not significant).*

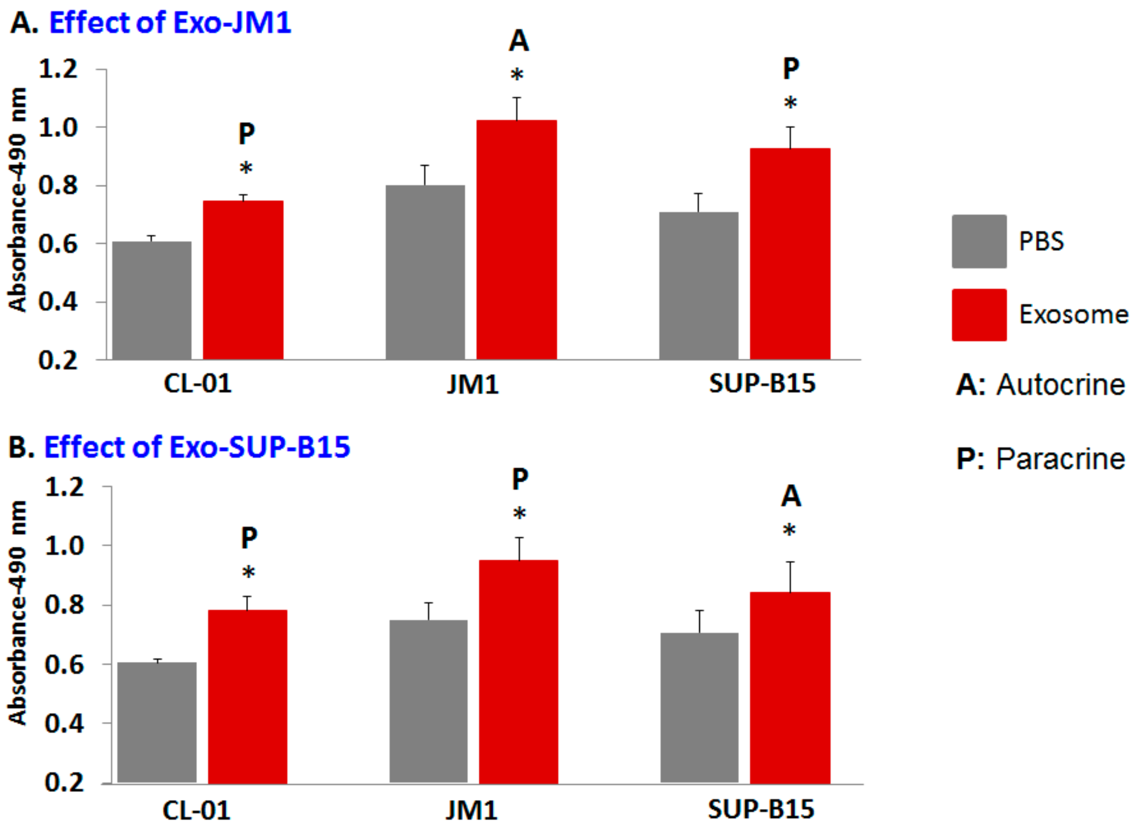

Figure S2. Exosomes induced cell proliferation by MTS assay. (A) CL-01, JM1, and SUP-B15 cells were plated (0.1x10^6/well) in quadruplets. Exo-JM1 (JM1 cell-derived exosomes) was loaded (250 µg/ml) on the CL-01, JM1, and SUP-B15 for 24 hours. Next day, MTS were added into the culture plate and plate was read at 490 nm. (B) CL-01, JM1, and SUP-B15 cells were plated (0.1x10^6/well) in quadruplets. Exo-SUP-B15 (SUP-B15 cell-derived exosomes) was loaded (250 µg/ml) on the CL-01, JM1, and SUP-B15 for 24 hours. Next day, cell proliferation was quantitated by MTS. Data was analyzed by PBS as control (P value * p<0.05, **p<0.01, ***p<0.001. NS; not significant).

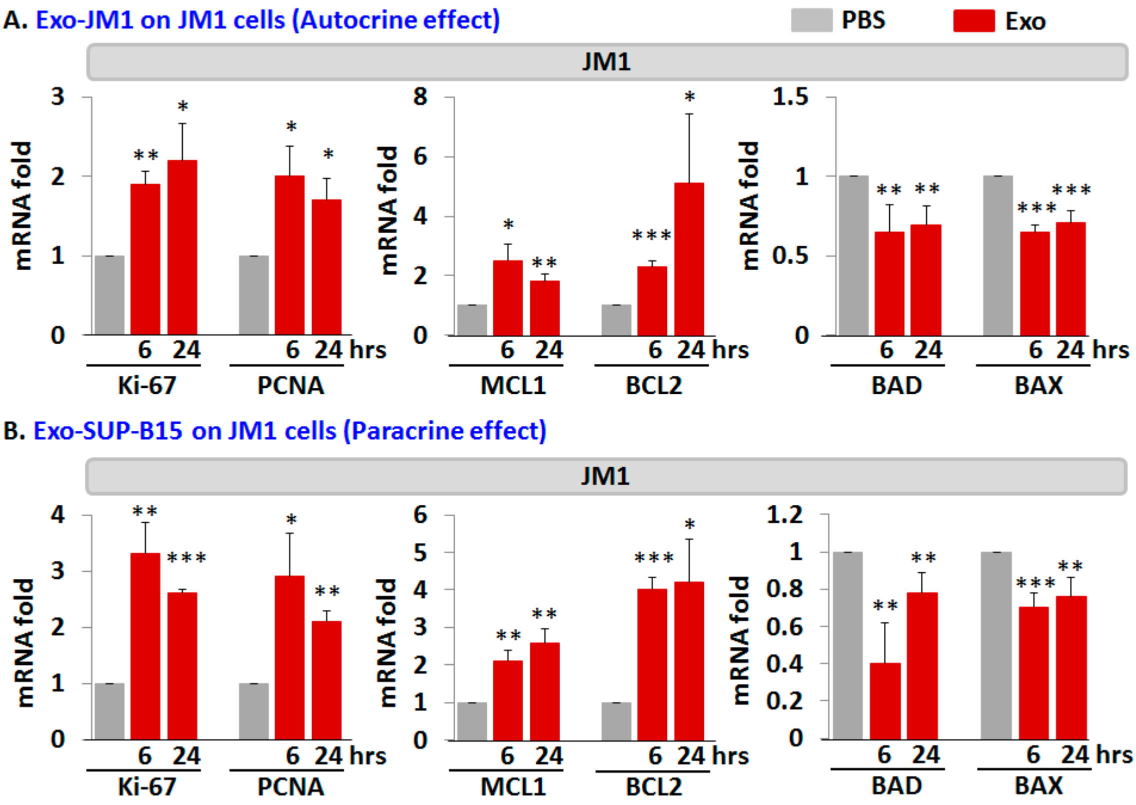

Figure S3. Exo-CM (JM1 and SUP-B15) regulates proliferative, pro-survival, and pro-apoptotic genes. (A) JM1 cells were exposed with Exo-JM1 (250 µg/ml) and cultured cells were harvested for RNA isolation at 6 hours and 24 hours post treatment. Indicated mRNA (Ki-67, PCNA, MCL1, BCL2, BAD, BAX) expression analyzed by q-PCR. (B) JM1 cells were exposed with Exo-SUP-B15 (250 µg/ml). Cells were harvested for RNA isolation at 6 and 24 hours after treatment. Indicated mRNA (Ki-67, PCNA, MCL1, BCL2, BAD, BAX) expression analyzed by q-PCR. Data represented are mean of three experiments. *(Ctrl: PBS only/no exosomes- P value *p<0.05, **p<0.01, ***p<0.001).*

Table S1. List of healthy donors and PALL serum samples.

Healthy Donor #	Serum Sample Code
1	HD77
2	HD78
3	HD79
4	HD80
**PALL patient #**	**Serum Sample Code**
1	PALL01 D1
	PALL01 D29
2	PALL02 D1
	PALL02 D29
3	PALL14c relapse
	PALL14c 2nd remission
4	PALL24 relapse
	PALL24 2nd remission
5	PALL14 relapse
	PALL14 2nd remission
6	PALL25 D1
7	PALL03
8	PALL04
9	PALL05
10	PALL05b
11	PALL14a

Table S2. Human primers from universal probe library (UPL).

Genes	Accession #	Probe #	Primers sequences
PCNA	J04718.1	77	For: 5'-CTTTTTCGCGCCAAAGTC-3'
			Rev: 5′-CTGCGGAAAAACCCTTGAT-3'
Ki-67	NM_002417.4	53	For: 5'-CGCGTAAGTCAAGACCAAAAT-3'
			Rev: 5'-GGTCAAGCTCTTGTTCAGGTG-3'
BAD	AF031523.1	45	For: 5'-ACCAGCAGCAGCCATCAT-3'
			Rev: 5'-GGTAGGAGCTGTGGCGACT-3'
BAX	U19599.1	55	For: 5′-CAAGACCAGGGTGGTTGG-3'
			Rev: 5'-CACTCCCGCCACAAAGAT-3'
MCL1	AF118124.	4	For: 5′-AAGCCAATGGGCAGGTCT-3'
			Rev: 5′-TGTCCAGTTTCCGAAGCAT-3'
BCL2	AY220759.1	23	For: 5′-TTGGTATCCTTCTCTTTCAGCAC-3′
			Rev: 5'-ATGGCATTGACGAAGAGGAT-3'
GAPDH	NM_002046.3	60	For: 5′-AGCCACATCGCTCAGACAC-3′
			Rev: 5′-GCCCAATACGACCAAATCC-3′
